# Entrapped Catheter across the Fossa Ovalis in an Adult with Pulmonary Stenosis - A Case Report of Surgical Relief

**Published:** 2014-01-01

**Authors:** Vithalkumar Malleshi Betigeri, Girish Gopinathan, Indira Malik, Manoj Kumar Sanwal, Vishnu Datt, Deepak Kumar Satsangi

**Affiliations:** 1 Department of Cardiovascular and Thoracic Surgery, GB Pant Hospital, New Delhi, India; 2 Department of Anesthesia, GB Pant Hospital, New Delhi, India

**Keywords:** Valvular Heart Disease Congenital, Pulmonary Valve Stenosis, Echocardiography, Cardiac Valve Annuloplasty, Complications

## Abstract

Percutaneous pulmonary balloon valvuloplasty as a procedure of choice in adults has been established since the last three decades. Even though the complications are rare, they are scarcely reported in the literature. We report such a case in an adult female patient of severe pulmonary valular stenosis in whom, entrapped catheter across the fossa ovalis was noted in chest x-ray and echocardiogram following unsuccessful percutaneous pulmonary balloon valvuloplasty. Our case emphasizes this rare complication and its successful surgical outcome.

## 1. Introduction

Congenital pulmonary valular stenosis constitutes 8 - 10% of all congenital heart disease (1). To alleviate the need for surgical valvotomy that was introducedin 1947 and with excellent short and long term results of Pulmonary Balloon Valvuloplasty (PBV) first reported by Semb et al. in 1979 in pediatric patients, PBV is also being applied in adult patient groups with excellent success rate and little known complications. We report a case of 30 year old female in whom, a catheter that was used during percutaneous PBV to treat her severe pulmonary stenosis was found broken and lodged across the inter atrial septum. Thus, she underwent a successful surgical intervention.

## 2. Case Presentation

**Figure 1.**
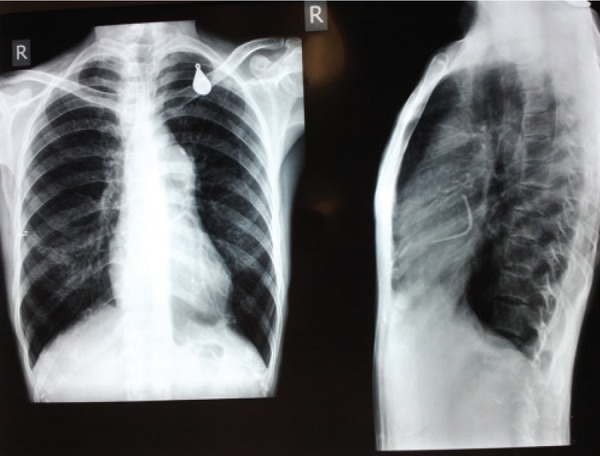
Chest X – Ray Posterior - Anterior and Lateral Views Showing Dilated Main Pulmonary Artery and the Catheter

**Figure 2.**
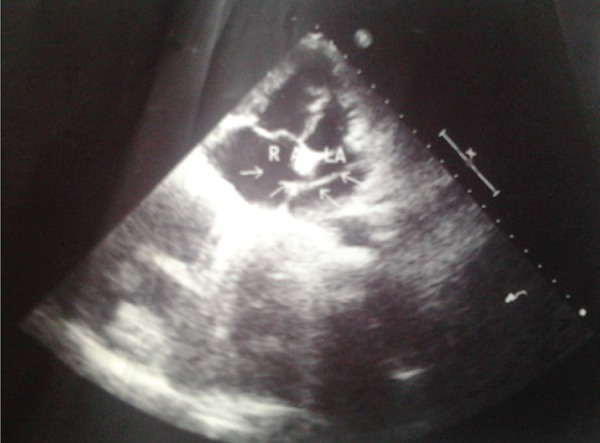
Echocardiogram Showing the Catheter Traversing the Interatrial Septum Abbreviations: RA, Right Atrium; LA, Left Atrium

A 30 year old female with diagnosis of severe pulmonary valvular stenosis for symptoms of on and off dyspnea on exertion and palpitations for 15 years duration had undergone unsuccessful percutaneous PBV. She was afebrile with heart rate of 70 / min, normal sinus rhythm, blood pressure of 108 / 68 mmHg, and peripheral saturation of 86%. She had grade IV / VI systolic murmur over the left sternal border, decreased P2, and raised JVP with palpable hepatomegaly. Besides, ECG was in normal sinus rhythm with evidence of right ventricular hypertrophy. Her chest x-ray revealed marked enlargement of the main pulmonary trunk with the catheter which appeared to lie in the left heart (Figure 1). Moreover, transthoracic echocardiography revealed severe pulmonary valve stenosis with Right Ventricular (RV) wall hypertrophy, peak systolic gradient of 76 mmHg across the Right Ventricular Outflow Tract (RVOT), the catheter traversing the interatrial septum (Figure 2), severe Tricuspid Regurgitation (TR), right ventricular dysfunction, mild pulmonary regurgitation, and normal left ventricular function. Intraoperative transesophageal echocardiography (TEE) confirmed the presence of a catheter lodged in the interatrial septum, severe TR, and peak gradient of 62 mmHg across the narrow hypertrophic RVOT.

**Figure 3.**
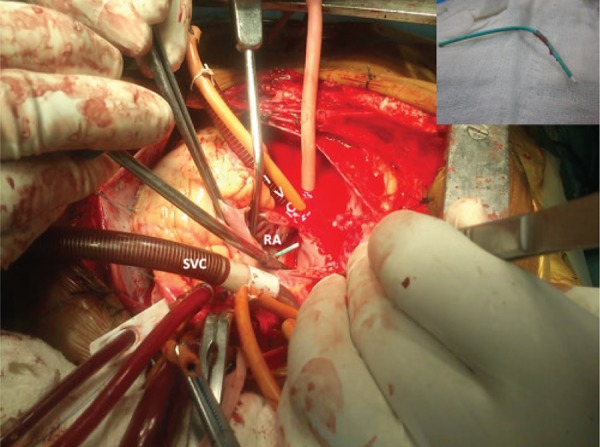
Intraoperative Photograph of the Entrapped Catheter in the Interatrial Septum Abbreviations: RA, Right Atrium; SVC, Superior Venacave; IVC, Inferior Venacave (Inset-extracted catheter tip)

**Figure 4.**
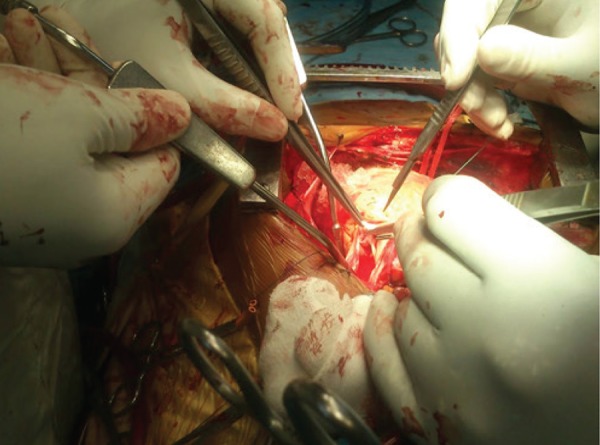
Intraoperative Photograph of Domed, Stenosed Bicuspid Pulmonary Valve

Intraoperative prebypass gradient across the RVOT was 63 mmHg. Under cardiopulmonary bypass and antegrade blood cardioplegic arrest, right atriotomy revealed that the catheter tip was entrapped within an area of fossa ovalis (Figure 3) away from a Patent Foramen Ovale (PFO). In addition, pulmonary arteriotomy showed (Figure 4) stenotic, bicuspid, thickened, and noncalcific pulmonary valve that allowed only Hegars dilator No. 16. Surgical pulmonary valvotomy along with infundibular myomectomy allowed No. 20 Hegars dilator freely. Gluteraldehyde treated pericardium was used to reconstruct RVOT and close iatrogenic atrial septal defect created by the catheter and the PFO. Moreover, modified De Vega’s tricuspid valve annuloplasty was carried out for dilated tricuspid valve annulus with morphologically normal leaflets which showed severe noncoaptation on the saline test. The patient was gradually weaned off bypass with moderate inotropic support of dobutamine 5 µg / kg / min and adrenaline 0.02 µg / kg / min. Post bypass gradient across RVOT was 22 mmHg. The patient had uneventful recovery with extubation after 24 hours and inotropic tapering over the next 48 hours. Finally, she was discharged on the tenth post operative day. After 6 months, postoperative transthoracic echocardiogram showed mild tricuspid and pulmonary regurgitation, no residual shunt, and RVOT gradient of 30 mmHg.

## 3. Disscussion

Percutaneous PBV as a procedure of choice in adults has revealed excellent results since the application of this procedure to adult congenital pulmonary stenosis by Pepine et al. (2) following the successful and excellent short and long term results of PBV in infants and children. Recent guidelines have recommended this procedure for a peak systolic gradient above 40 mmHg in neonates with critical stenosis or in significant obstruction in the face of RV dysfunction. So far, various complications have been reported in the literature during PBV procedure, including arrhythmias, valvular injury, pulmonary regurgitation, pulmonary artery dissection, perforation of distal pulmonary vessels, RVOT perforation (3, 4), embolic manifestations, acute pulmonary edema (4), acute pulmonary reperfusion hemorrhage (5), and development of infundibular obstruction (6). There is a consensus regarding myomectomy of post procedural RV gradient above 80 mmHg which may be due to significant infundibular stenosis, a problem that causes high residual pressure gradient after PBV (7).

Less severe pulmonary valve stenosis does not often have the combination of valvular and infundibular components. Occurrence of increased infundibular gradient with hyperdynamic right ventricle and decreased forward flow through the right ventricular out flow is a known complication following successful relief from severe pulmonary valve stenosis by PBV. Up to now, no studies have quantified the occurrence of such complications, level of RV pressure, and severity of pulmonary stenosis to induce such a complication (8, 9).

Presence of PFO here acted as a safety valve to decompress the failing heart to maintain adequate cardiac output. RV dysfunction and severe tricuspid regurgitation were taken care of by operative procedure quite well as was the successful removal of the entrapped catheter tip along with the relief of RVOT obstruction. Even though the exact cause of this accident could not be confirmed in our case, using reused balloon catheters as a cause cannot be ruled out since in developing countries cardiologist use reused sterilized catheters as an economically viable option (10). Moreover, as the catheter entrapped in the fossa ovalis could not possibly be retrieved with a snare in the cath lab, it became mandatory that the patient has to undergo surgery. The open heart surgery dislodged the retained catheter and also took care of associated lesions.

Our case highlighted the successful surgical relief of failing right ventricle with severe tricuspid regurgitation in an adult female patient with severe pulmonary valve stenosis along with extraction of entrapped fragment of the catheter tip after unsuccessful percutaneous PBV.
